# Mechanism of Chronic Stress-Induced Glutamatergic Neuronal Damage in the Basolateral Amygdaloid Nucleus

**DOI:** 10.1155/2021/8388527

**Published:** 2021-11-23

**Authors:** Songjun Wang, Xia Liu, Weibo Shi, Qian Qi, Guozhong Zhang, Yingmin Li, Bin Cong, Min Zuo

**Affiliations:** Hebei Key Laboratory of Forensic Medicine, Collaborative Innovation Center of Forensic Medical Molecular Identification, Department of Forensic Medicine, Hebei Medical University, Shijiazhuang, China

## Abstract

Stress is a ubiquitous part of our life, while appropriate stress levels can help improve the body's adaptability to the environment. However, sustained and excessive levels of stress can lead to the occurrence of multiple devastating diseases. As an emotional center, the amygdala plays a key role in the regulation of stress-induced psycho-behavioral disorders. The structural changes in the amygdala have been shown to affect its functional characteristics. The amygdala-related neurotransmitter imbalance is closely related to psychobehavioral abnormalities. However, the mechanism of structural and functional changes of glutamatergic neurons in the amygdala induced by stress has not been fully elucidated. Here, we identified that chronic stress could lead to the degeneration and death of glutamatergic neurons in the lateral amygdaloid nucleus, resulting in neuroendocrine and psychobehavioral disorders. Therefore, our studies further suggest that the Protein Kinase R-like ER Kinase (PERK) pathway may be therapeutically targeted as one of the key mechanisms of stress-induced glutamatergic neuronal degeneration and death in the amygdala.

## 1. Introduction

Stress induces the activation of the body's adaptive response system, which plays a critical role in modulating human behavior in all walks of life. In today's fast moving world, stress has become an inevitable and/or integral part of our life. Interestingly, the moderate stress level has been shown to enhance the general adaptability of the body with respect to physical, mental, and emotional performances [1]. However, persistent and mismanaged stress can lead to chronic and irreversible injury of the body, such as life-threatening cardiovascular dysfunction [2, 3], progressive neurodegeneration [4], and even cancer [5]. Moreover, emerging studies have confirmed that persistent mental stress can also lead to pathological behavioral changes irrespective of aging [6–8].

As a key component of the limbic system, the amygdala plays a crucial role in the onset and progression of stress-induced phobic anxiety and affective disorders [9, 10]. The amygdala is mainly divided into basolateral complex, central nucleus, medial nucleus, and cortical nucleus. Lateral nuclei of the basolateral complex serve as the main sensory information input system to the central nucleus of the amygdala by sending out the processed information that is received from the prefrontal auditory cortex, hippocampus, and interconnected accessory parts. Processed and integrated information relayed to the central nucleus then modulate the function of the hypothalamic-pituitary-adrenocortical (HPA) axis towards emotional arousal [11, 12].

Previous studies have confirmed that the basolateral amygdala (BLA) plays an important role in fear learning and memory and is a key structure in the neural fear circuit which has been widely concerned [13]. BLA consists of 80% of excitatory neurons (glutamatergic neurons) and 20% of inhibitory neurons (GABAergic neurons) [14]. Previous studies have found that acute stressors can induce the high expressions of glutamate (Glu) receptors in the BLA nucleus, which then gradually get adjusted to low expression steady-state level in response to prolonged stress exposure through a yet unknown mechanism [15].

Stress leads to activation of the amygdala and simultaneously induces a large increase in the excitatory neurotransmitter glutamate pool, which plays an important role in neural regulation. Studies have shown that the toxic effects of excitatory neurotransmitters can also cause both acute and chronic nerve cell injuries [16, 17]. However, the underlying mechanism of chronic stress-induced excitotoxicity remains enigmatic. The endoplasmic reticulum (ER) is the main site for protein synthesis, lipid production, and calcium storage in eukaryotic cells and is important for cellular functions and survival. While moderate ER stress can relieve ER pressure and may promote the recovery of its normal physiological functions, chronic excessive ER stress can provoke metabolic disorders and degenerative mechanisms leading to apoptotic cell death [18, 19].

In this study, we have established an experimental stress model in rats to investigate the pathomechanism of stress-induced behavioral changes. Furthermore, we investigated how functional modulation of BLA might be related to the ER stress, protein kinase-like ER kinase (PERK) pathway, and excitotoxicity of glutamatergic neurons in the amygdala. Our results thus illustrate the morphological basis for the chronic stress-induced glutamatergic neuronal damage in the BLA and its relationship with the underlying pathomechanism.

## 2. Materials and Methods

### 2.1. Experimental Animals

Animal experiments were performed in accordance with the experimental animal research program approved by the laboratory animal management committee. Adult male Sprague-Dawley (SD) rats (Experimental Animal Center, Hebei Medical University, China), weighing 220 ± 20 g, were bred in the experimental environment for 7 days. During the adaptive feeding period, the rats were given grasping and touching every day to reduce the degree of basic stress during experimental procedures. According to the principle of random grouping, the experimental animals were divided into the control and stress groups (restraint stress combined with ice water swimming) at 1, 3, 7, 14, and 21 days' timepoints with 10 rats in each group. In order to study how the ER stress affects glutamatergic neurons of the BLA under stress exposure, we enrolled a group treated with the PERK pathway inhibitor Sal after stress exposure (stress+Sal) and another group with only Sal treatment for 14 days (Sal; *n* = 10 rats in each group).

### 2.2. Preparation of Stress Model (Restraint+Ice Water Swimming)

The experimental stress model of a rat was established according to previous studies [15, 18, 19]. They were able to stretch their legs, but with the restrainers, they could not move for more than 6 hours at once. The stress process lasted for 1, 3, 7, 14, or 21 days. Stress experiments were carried out in a separate and independent room, and the rats in the control group did not eat or drink water at the same time. Rats in the stress+Sal group were injected intraperitoneally (i.p.) with Sal (1 mg/kg, i.p.) 30 minutes prior to stress treatment. Rats in the Sal group were injected with Sal (1 mg/kg i.p.) only. The protocol was performed for 14 days for both groups.

### 2.3. Behavioral Experiment

#### 2.3.1. Open Field Experiment

This test mainly examines the exploratory as well as anxiety behavior of experimental animals in the new environment [20]. The open field, made of odorless steel material in a square shape, was placed in a room with proper sound insulation. The length, width, and height of the open field were 90 cm × 90 cm × 30 cm, respectively. The inner surface was painted jet black. The bottom of the inner side was distributed into 16 squares with the same area by a 6 mm wide white line. The movement track of rats was recorded by a camera. The experiment lasted for 5 minutes, and the four small squares in the center were set as the central area and the rest as the marginal area. After the completion of each rat experiment, the open field was scrubbed with 75% ethanol, and the next animal experiment was conducted after drying and checked odorless. Total moving distance, central area movement distance, and central area movement time were utilized to measure the anxiety-like behavior in these rats. The less the distance and time of moving into the central area, the higher was the anxiety level of rats and vice versa.

#### 2.3.2. Elevated plus Maze Experiment

The experimental devicewas consisted of a central area (10 cm × 10 cm), two relatively closed arms (50 cm × 40 cm × 10 cm), and two relatively open arms (50 cm × 10 cm) and was placed 50 cm away from the ground. The rats were put into the open arms from the central grid, and their activity tracks were recorded by an electronic pathway system within 5 minutes. After the completion of each rat experiment, the maze was scrubbed with 75% ethanol, and the next animal experiment was conducted after drying and checked odorless. Residence time and displacement of open arm and closed arm were utilized to measure the anxiety-like behavior in these experimental rats. The less the stay time and displacement of the open arm, the higher was the anxiety level of rats and vice versa [21, 22].

### 2.4. Tissue Preparation

The rats were deeply anesthetized before sacrifice, one hour after the behavioral test. The harvested brains were immediately fixed in 4% paraformaldehyde solution. According to the stereotactic map of the rat brain [23], the brain tissue was cut at -1.80 mm from the anterior fontanelle and then dehydrated in the graded ethanol series and immersed in paraffin; then, sections of 5 *μ*m thickness were prepared for H&E staining, FJB staining, and IHC analysis. Brain tissues for neurotransmitter analysis were quickly removed and stored at −80°C. BLA was cut according to the stereotactic map of the rat brain to measure the Glu content.

### 2.5. Estimation of Glu Levels in the BLA

Using HPLC and fluorescence detector [24], the content of Glu in tissues was measured. The BLA (10 mg) sample was homogenized in saline (1 g: 9 ml). 20 *μ*l of BLA homogenate was mixed with 70 *μ*l of 0.4 M perchloric acid and centrifuged at 12000 xg and 4°C for 20 minutes. The supernatant was removed for analysis and filtered with a 0.22 mm GV filter (Millipore, Bedford, MA, USA). After that, using a temperature-controlled automatic injector (Agilent), 10 *μ*l of each sample was injected into the Agilent borohill HPH-C18 column (4.6 mm × 50 mm, 2.7 *μ*m). The mobile phase consisted of 10 mM sodium hydrogen phosphate buffer and 10 mM sodium borate buffer solution in water, methanol, and acetonitrile (78: 13 : 9, *v*/*v*). The flow rate was constantly kept at 1 ml·min^−1^. The chromatographic analysis was carried out at 35°C. The working wavelength of the fluorescence detector was 355 nm, and the emission wavelength was 450 nm. Methanol was adopted as an internal standard, and HP ChemStation software (Agilent, Santa Clara, USA) was used to quantify the Glu level.

### 2.6. Fluoro-Jade B (FJB) Staining

Brain tissue sections were dewaxed and incubated in 0.06% potassium permanganate solution for 10 minutes (room temperature, shaking table). The treated slices were incubated in 0.0001% FJB (Millipore, USA, product No. AG310, batch number NG2730022) for 10 minutes (room temperature), and strong light exposure was avoided during the reaction. After being sealed with neutral gum, sections were observed and photographed under a fluorescence microscope.

### 2.7. Immunohistochemistry (IHC)

Following the IHC kit manufacturer's protocol, the paraffin sections were pretreated by heat-treated antigen recovery method and then incubated in a solution of 3% hydrogen peroxide in methanol for 30 minutes, then in goat serum for 30 minutes. Next, the tissue sections were incubated overnight at 4°C with antibodies against p-eIF2*α* (Cat. Ab32157, 1 : 200, Abcam, Cambridge, MA, United States), ATF4 (Cat. ab221971, 1 : 300, Abcam, Cambridge, MA, United States), and CHOP (Cat. ab233121,1 : 300, Abcam, Cambridge, MA, United States). The tissue samples were incubated for 1 hour with a biotinylated secondary antibody and then with horseradish peroxidase for 30 minutes. 3,3′-Diaminobenzidine (DAB) was utilized as the chromogen to detect the target proteins. The tissue sections were then counterstained with hematoxylin.

### 2.8. Cell Counting

Morphological observation and data analysis were performed on 5 rats in each group. According to the stereotactic map, the BLA region of the amygdala was excised. To quantify DAB-positive cells, the microscope-based polychromatic histocytology (MMTC) has been universally used [25]. Using a tissue analysis system (tissue Gnostics, Vienna, Austria) of Zeiss Axioimager z2 microscope (Jena, Germany) in the 100x field of vision, we obtained the corresponding images, delineated the area of the amygdala BLA, and analyzed the neurons in this area with HistoQuest software. HistoQuest staining software was used as a tool to identify the types of surrounding cells and the cell structure by hematoxylin staining. In this study, the brown shadow formed by DAB staining and hematoxylin staining were automatically identified and separated by the system through a light intensity difference. HistoQuest software was used to quantitatively analyze the average intensity of glutamatergic neurons in IHC staining in each section of BLA. The original data were imported into SPSS 21.0 (IBM, Armonk, NY, USA) for further analysis.

For FJB fluorescence staining analysis, each section in the amygdala region was randomly selected 200 times for the bright field-related counting work, and two observers observed the average number after counting.

### 2.9. Statistical Methods

By Kolmogorov-Smirnov test, the distribution was normalized (*P* > 0.1). Results were expressed as mean ± SEM. Since all samples were normally distributed, one-way ANOVA was used for statistical analysis. The action of the inhibitor Sal was analyzed by two-way ANOVA. When the comparison was limited to two experimental groups, Student's *t*-test was used. All statistical analyses were carried out utilizing GraphPad Prism 5 (GraphPad Software Inc., San Diego, CA, USA) and SPSS 21.0. The statistically significant threshold was *P* < 0.05.

## 3. Results

### 3.1. Stress Leads to Anxiety-Like Behavior

Both open field experiment and elevated plus maze test are standard and well-established methods to examine anxiety-like behaviors in rodents.

**Figure 1:**
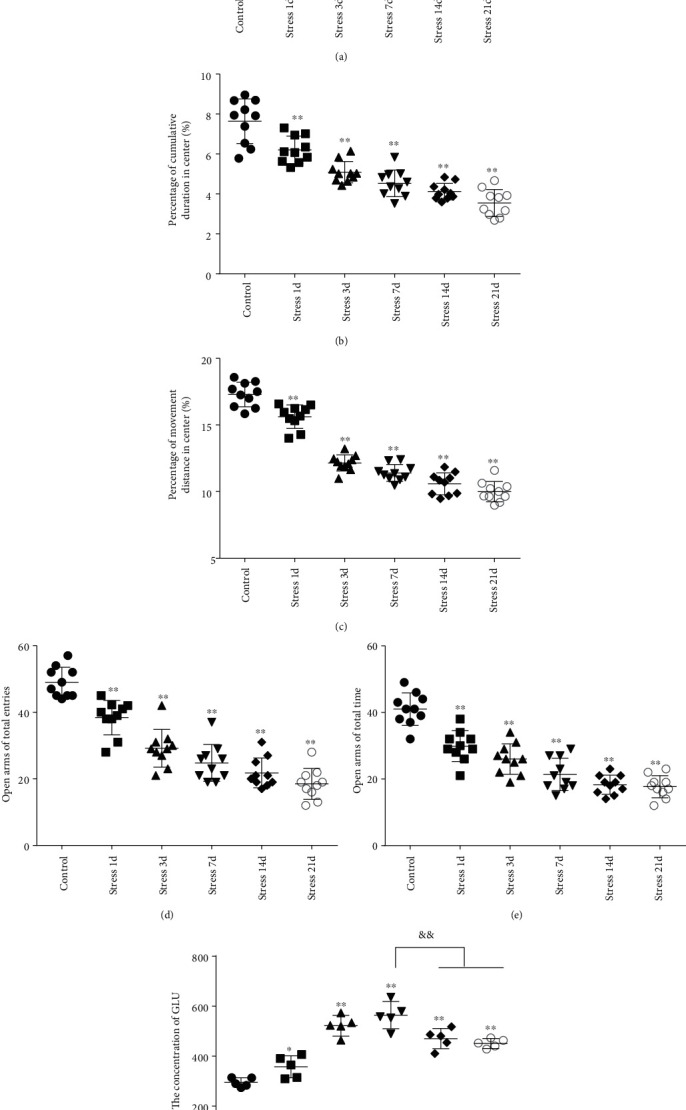
Stress caused anxiety-like behavior and Glu level changes in the BLA of rats. (a) Total movement distance in the open field test. (b) Percentage of cumulative duration in the center area of the open field test. (c) Percentage of movement distance in the center area of the open field test. (d) Percentage of open arm entries on the elevated plus-maze (EPM). (e) Percentage of time spent in open arms of the EPM. (f) The concentration of Glu in the BLA. Data are shown as mean ± SEM; ^∗^*P* < 0.05, ^∗∗^*P* < 0.01 vs. the control group (*n* = 10). ^&&^*P* < 0.01 vs stress 7 d.

Open field experiment revealed that stress could induce notable changes in the central area exercise time ratio (*F* = 44.103, *P* < 0.01) and the central area exercise distance ratio (*F* = 142.526, *P* < 0.01), without affecting the movement ability of the study animals (*F* = 0.824, *P* > 0.05) as measured by one-way ANOVA. As demonstrated in Figures 1(a)–1(c), compared with the control group, the total activity-related distance traveled in each group was not significantly different, indicating that there was no difference in the motor ability in those rats. However, the central area exercise time ratio (*P* < 0.05), and the central area exercise distance ratio (*P* < 0.05) in the stress group were significantly reduced (*P* < 0.05) compared with that of the control group rats.

The elevated plus maze test exhibited that the percentage of open arm entries (*F* = 52.361, *P* < 0.01) and the percentage of time spent in open arms (*F* = 4.371, *P* < 0.01) were significantly altered after stress exposure, as measured by ANOVA. Figures 1(d) and 1(e) show that compared with the control group outcomes, the percentage of open arm entries (*P* < 0.05) and the percentage of time spent in open arms (*P* < 0.01) were significantly reduced (*P* < 0.01) in the stress group, further suggesting that stress exposure may lead to anxiety in rats.

### 3.2. Stress Perturbs the Content of Glu in the BLA

The content of Glu in the BLA was measured by high-performance liquid chromatography (HPLC) analysis. ANOVA revealed that the Glu contents in the BLA were significantly changed (*F* = 34.715, *P* < 0.01) in stress-induced rats. As shown in Figure 1(f), Glu content was rapidly increased following the stress exposure (357.39 ± 43.95), reaching a peak on the 7^th^ day of stress exposure (564.60 ± 54.56). Notably, the content of Glu exhibited a significant decrease on the 14^th^ day (470.47 ± 40.31) and 21st day (450.94 ± 19.75) compared with that on the 7^th^ day (*P* < 0.01), but still was at the increased level with respect to the control group (295.46 ± 18.46, *P* < 0.01).

### 3.3. Stress Exposure Damages Glutamatergic Neurons

In the BLA, more than 80% of neurons are glutamatergic neurons, owing to its characterization as a large nucleus with abundant cytoplasm, which makes them easy to distinguish from GABAergic neurons and glial cells. We used hematoxylin and eosin (H&E) and a high-affinity Fluoro-Jade B (FJB) staining to investigate the stress-induced glutamatergic neuronal degeneration in the BLA.

**Figure 2:**
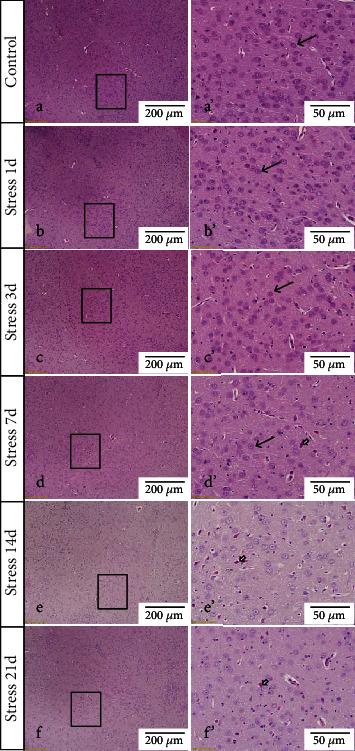
Stress leads to pyknosis of neurons in the BLA of rats. Representative images showing H&E staining in the BLA (a–f). (a′–f′) Magnified areas of (a–f). Glutamatergic neurons are shown by the black arrows; pyknosis of glutamatergic neurons is shown by the hollow arrows. Bars = 200 *μ*m in (a–f); bars = 50 *μ*m in (a′-f′).

The H&E staining in Figure 2 revealed that the structure of glutamatergic neurons was normal in the control group. But at 1-day and 3-day timepoints following stress exposure, the tissue structure was slightly loosened, and glutamatergic neurons were found with edema. However, with prolonged stress exposure, some degenerative dead cells were also observed at 7 days, while more degenerated and pyknotic neurons were detected at 14-day and 21-day poststress exposure.

**Figure 3:**
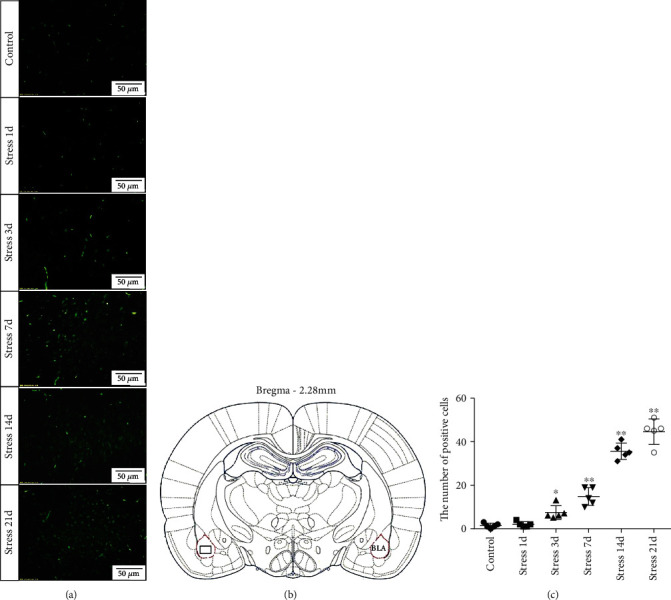
Stress leads to degeneration and death of neurons in the BLA of rats. (a) Representative images are showing Fluoro-Jade B staining in the BLA. The positive cells were labeled with green fluorescence. Bars = 50 *μ*m. The analyzed region within the BLA is schematically illustrated in (b) (diagram modified from Paxinos and Watson, 2007). (c) Quantitative analysis of the positive cells number in the BLA. Data are shown as mean ± SEM; ^∗^*P* < 0.05, ^∗∗^*P* < 0.01 vs. the control group (*n* = 5).

FJB staining in Figure 3(a) showed that the degenerated and dead neurons were specifically stained in green [26]. ANOVA revealed that there was a significant variation in the degeneration and death of glutamatergic neurons in the BLA of stress-exposed rats (*F* = 128.678, *P* < 0.01). Compared with that of the control group, the degeneration and death of glutamatergic neurons were more prominent at 3 days of stress exposure, and the number of glutamatergic neurons was gradually increased with prolonged stress duration.

### 3.4. Inhibition of the PERK Pathway Alleviates Anxiety Behavior in Rats

Previous studies have shown that the ER-PERK pathway is involved in stress-induced neuronal injury. To test whether it was also involved in the stress-induced injury of glutamatergic neurons in the BLA, experimental rats were treated with the ER stress inhibitor salubrinal (Sal).

**Figure 4:**
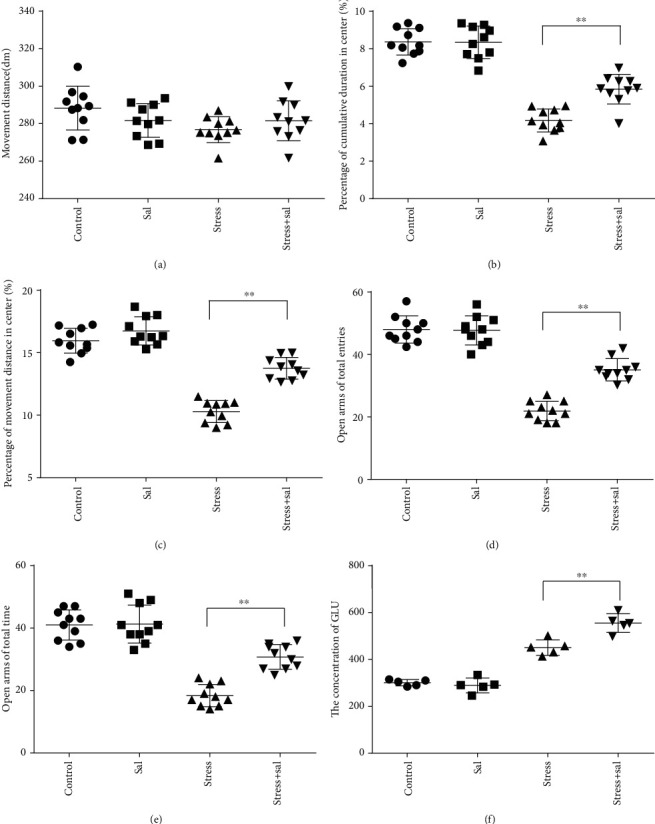
Inhibition of the PERK pathway alleviated anxiety-like behavior and improved Glu level in the BLA of rats. (a) Total movement distance on the open field test. (b) Percentage of cumulative duration in the center area of the open field test. (c) Percentage of movement distance in the center area of the open field test. (d) Percentage of open arm entries on the EPM. (e) Percentage of time spent in open arms of the EPM. (f) The concentration of Glu in the BLA. Data are shown as mean ± SEM; ^∗^*P* < 0.05, ^∗∗^*P* < 0.01 vs. the control group (*n* = 10).

In the open field test, as shown in Figures 4(a)–4(c), two-way ANOVA demonstrated that there was significant variation in interaction between stress exposures with and without Sal treatment on the cumulative duration in the center area (*F* = 199.569, *P* < 0.001; *F* = 12.144, *P* = 0.001) and the percentage of movement distance in the center area (*F* = 197.981, *P* < 0.001; *F* = 47.761, *P* < 0.001), while no remarkable effect on the total movement distance was detected (*F* = 3.59, *P* = 0.066; *F* = 0.091, *P* = 0.765). As demonstrated in this experiment, compared with the stress-exposed group, the Sal treated and stress-exposed (stress+Sal) rats exhibited significant increase in the percentage of cumulative duration in the center (*F* = 12.709, *P* = 0.001) and the percentage of movement distance in the center (*F* = 18.855, *P* < 0.01). Consistently, in the elevated plus maze test, as shown in Figures 4(d) and 4(e), two-way ANOVA showed that there was significant variation in interaction between stress exposures with and without Sal treatment on the percentage of open arm entries (*F* = 234.87, *P* < 0.001; *F* = 26.097, *P* < 0.001) and the percentage of time spent in open arms (*F* = 124.172, *P* < 0.001; *F* = 18.28, *P* < 0.001). Likewise, compared with the stress-exposed group, the percentage of time spent in open arms (*F* = 16.594, *P* < 0.001) and the percentage of open arm entries (*F* = 28.581, *P* < 0.001) in the stress+Sal group were significantly increased, suggesting that inhibition of the PERK pathway (or ER stress) may alleviate anxiety behavior.

### 3.5. PERK Inhibition Reduces Glutamatergic Neuronal Injury and Neurotransmitter

Given that mental and behavioral changes are closely related to the structure and function of the amygdala neurons, we measured the population of glutamatergic neurons in the amygdala with or without Sal treatment in stress-exposed rat brains. We also estimated the concentration of Glu in the amygdala using HPLC after Sal administration. Two-way ANOVA of Glu levels showed significant effects on the interaction among the stress+Sal treatment, stress only, and Sal treatment alone (*F* = 17.462, *P* = 0.001; *F* = 223.182, *P* < 0.001; and *F* = 11.238, *P* = 0.004). Post hoc test revealed that the levels of Glu exhibited significant improvement in Sal pretreated stress exposed rats (*P* < 0.01, *P* < 0.01, and *P* < 0.01; Figure 4(f)). Taken together, these results suggest that inhibition of the PERK pathways may improve the chances of neurotransmitter levels in the BLA.

**Figure 5:**
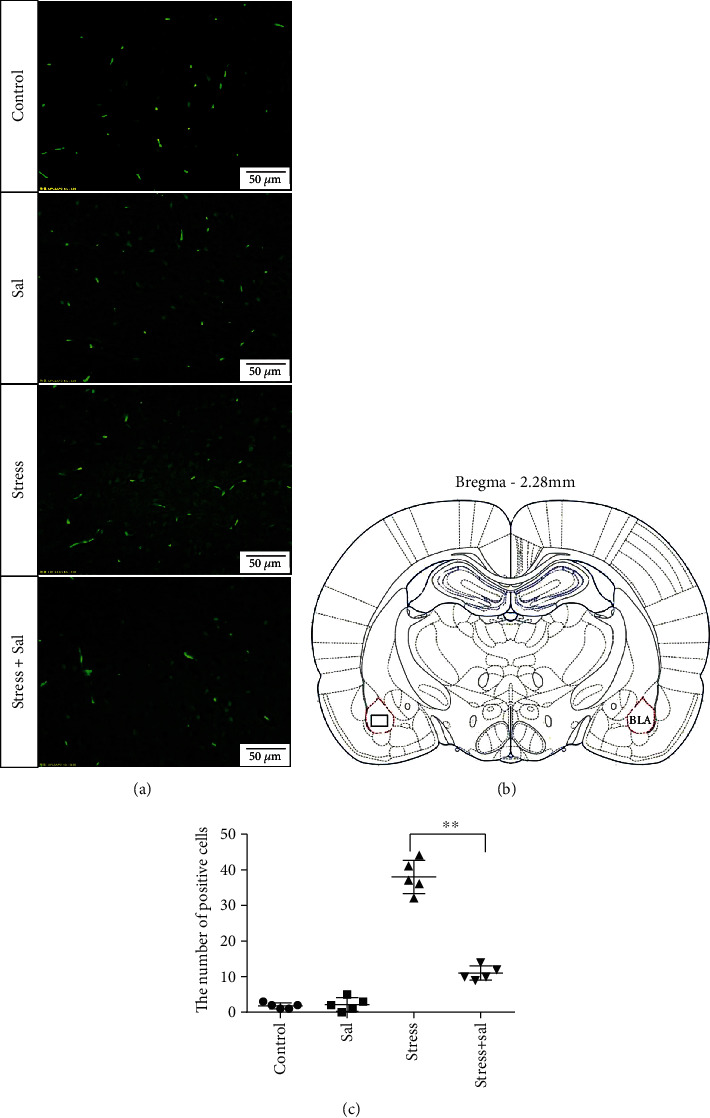
Inhibition of the PERK pathway alleviated degeneration and death of neurons in the BLA of stressed rats. (a) Representative images showing FJB staining in the BLA of rats. The positive cells were labeled with green fluorescence. Bars = 50 *μ*m. The analyzed region within the BLA is schematically illustrated in (b) (diagram modified from Paxinos and Watson, 2007). (c) Quantitative analysis of the FJB positive cell number in the BLA of rats (two-way ANOVA). Data are shown as mean ± SEM, ^∗∗^*P* < 0.01 vs. the stress group (*n* = 5).

Two-way ANOVA of FJB staining-positive (Figure 5) cells revealed significant effects on the stress treatment, Sal treatment, and the interaction between stress and Sal treatment (*F* = 334.925, *P* < 0.001; *F* = 114.256, *P* < 0.001; and *F* = 124.879, *P* < 0.001). The post hoc test showed there was an increase (*P* < 0.01) in the number of FJB staining-positive cells in the BLA after stress treatment. In contrast, stress+Sal treatment resulted in a decrease (*P* < 0.01) of the number of FJB staining-positive cells in the BLA.

### 3.6. Stress-Induced PERK Pathway Activation Damages Glutamatergic Neurons in the Amygdala

**Figure 6:**
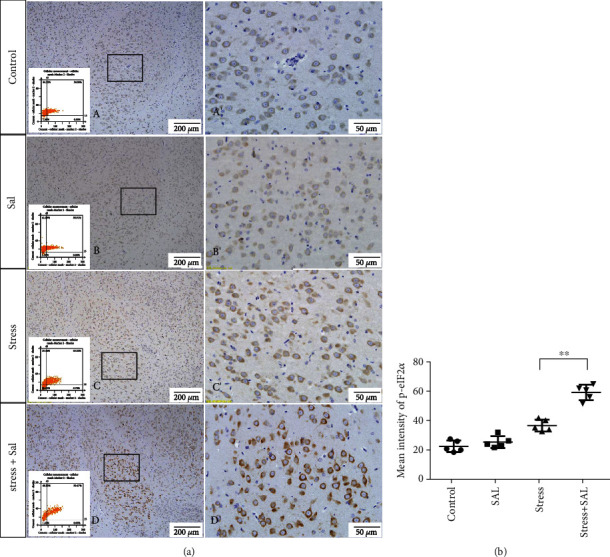
Inhibition of the PERK pathway alleviated the expression of p-eIF2*α* in glutamatergic neurons in the BLA of stressed rats. (a) Representative images are showing p-eIF2*α* immunohistochemistry in the BLA. Images obtained by MMTC are shown in the lower-left corners. (A′–D′) are magnified areas of (A–D). Bars = 200 *μ*m in (A–D). Bars = 50 *μ*m in (A′–D′). (b) Quantitative MMTC analysis. Data are shown as mean ± SEM; ^∗∗^*P* < 0.01 vs. the control group, ^∗^*P* < 0.05 vs. stress group (*n* = 5).

**Figure 7:**
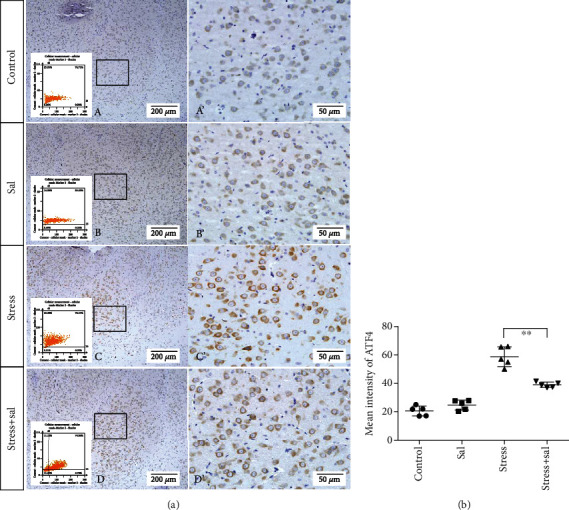
Inhibition of the PERK pathway alleviated expression of ATF4 in glutamatergic neurons in the BLA of stressed rats. (a) Representative images showing ATF4 immunohistochemistry in the BLA. Images obtained by MMTC are shown in the lower-left corners. (A′–D′) are magnified areas of (A–D). Bars = 200 *μ*m in (A–D); bars = 50 *μ*m in (A′–D′). (b) Quantitative MMTC analysis. Data are shown as mean ± SEM; ^∗∗^*P* < 0.01 vs. the control group; ^∗^*P* < 0.05 vs. the stress group (*n* = 5).

**Figure 8:**
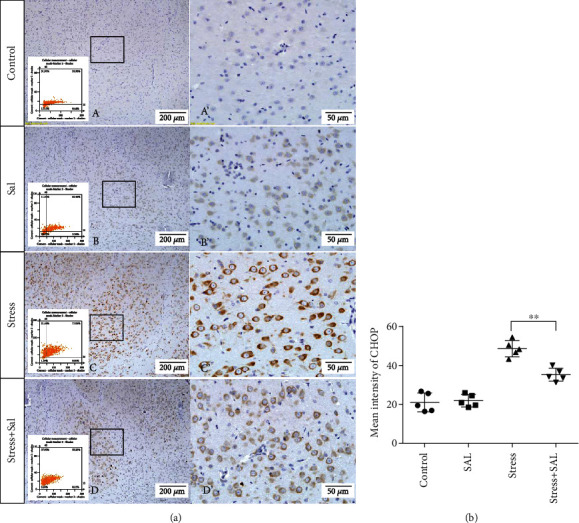
Inhibition of the PERK pathway alleviated expression of CHOP in glutamatergic neurons in the BLA of stressed rats. (a) Representative images are showing CHOP immunohistochemistry in the BLA. Images obtained by MMTC are shown in the lower-left corners. (A′–D′) are magnified areas of (A–D). Bars = 200 *μ*m in (A–D); bars = 50 *μ*m in (A′–D′). (b) Quantitative MMTC analysis. Data are shown as mean ± SEM; ^∗∗^*P* < 0.01 vs. the control group, ^∗^*P* < 0.05 vs. the stress group (*n* = 5).

The expressions of PERK pathway-related phosphorylated eukaryotic initiation factor 2*α* (p-eIF2*α*), activating transcription factor 4 (ATF4), and the C/EBP homologous protein (CHOP) was detected in the cytoplasm of glutamatergic neurons by immunohistochemical (IHC) staining (Figures 6–8).

HistoQuestr software was used to quantify the immunohistochemical mean intensity of glutamatergic neurons in the BLA.

Two-way ANOVA of p-eIF2*α*, ATF4, and CHOP immunohistochemical mean intensity values revealed significant effects on the stress only, Sal treatment alone, and stress+Sal treatment (p-eIF2*α*: *F* = 42.812, *P* < 0.001; *F* = 25.812, *P* < 0.001; *F* = 152.267, *P* < 0.001; ATF4: *F* = 180.475, *P* < 0.001; *F* = 15.95, *P* = 0.001; *F* = 37.503, *P* < 0.001; and CHOP: *F* = 133.954, *P* < 0.001; *F* = 12.424, *P* = 0.003; *F* = 18.169, *P* = 0.001). Post hoc test indicated that there was an increase in the immunohistochemical mean intensity of p-eIF2*α*, ATF4, and CHOP expressions in glutamatergic neurons after stress treatment (*P* < 0.01, *P* < 0.01, and *P* < 0.01). In contrast, Sal treatment alone and the stress+Sal treatment decreased (*P* < 0.01, *P* < 0.01) the immunohistochemical mean intensity of ATF4 and CHOP in the BLA-specific glutamatergic neurons.

## 4. Discussion

The BLA nucleus, the key structure of the brain for learning and memory of fear emotion adapts to stress response by regulating its neuroendocrine functions. However, under the sustained effects of overloaded stress levels, it can lead to neuroendocrine dysfunction and psychobehavioral disorders [27, 28]. Our established experimental stress model in rat, which can have both physical as well as psychological stress exposures, is an ideal model for stress-related pathomechanistic investigations. This study indicates that chronic stress exposure dramatically increases the Glu expression in the BLA nucleus leading to the emergence of anxiety-like behavior. Furthermore, the stress-induced changes in Glu content in the BLA nucleus may be the functional response of nerve cells and/or the functional cum structural changes of the amygdala-specific glutamatergic neurons. Therefore, a detailed understanding of the specific pathomechanism is worthy of further investigation.

Stress-triggered nerve impulses are transmitted to the excitatory neurons of the basolateral nucleus of the amygdala, resulting in a large amount of Glu secretion to maintain its corresponding functional state [29, 30]. However, under continuous and excessive stress exposure, the excitatory toxicity of Glu can cause damage to neurons or even induction of neuronal apoptosis [31, 32]. Neuroimaging studies in patients with stress disorder have found that the amygdala can be activated under stressed conditions, and its volume changes in response to stress level [33]. These results indicate that stress may lead to certain vivid changes in the morphology and function of the amygdala. In this study, H&E and FJB staining revealed that with the prolongation of stress duration, the number of degenerated and dead glutamatergic neurons in the BLA nucleus was gradually increased, indicating that chronic stress could lead to the degeneration of glutamatergic neurons in the BLA, especially after 7 days of stress exposure. The glutamatergic neuronal dysfunction in the early stage of stress exposure is mostly related to its functional changes, while that in the later stage of stress is caused by both functional as well as structural changes of the amygdala. Compared with H&E staining, FJB staining results more clearly showed that the number of degenerative and dead cells was significantly increased along with early onset. It could be because of the fact that FJB fluorescence staining is more sensitive than H&E staining. In agreement with the previous studies, our results provide morphological evidence that chronic stress can lead to degeneration and death of GABAergic neurons [15] and astrocytes [34], leading to the decrease of the amygdala volume and may be related to the degeneration and death of glutamatergic neurons in the BLA nucleus.

Stress leads to sustained and enhanced expression of Glu in the BLA nucleus. Excitatory neurotransmitter amino acids can induce a large amount of Ca^2+^ influx through N-methyl-D-aspartate (NMDA) receptor, resulting in intracellular Ca^2+^ overload, Ca^2+^ dyshomeostasis, perturbed ER function, and ER stress induction [35, 36]. In the early stage of the stress response, p-eIF-2*α* inhibits protein translation and synthesis, thereby reducing the load of protein folding at ER as a protective effect on stressed cells [37, 38]. But, with increasing stress duration and intensity, p-eIF-2*α* activates the expression of transcription factor ATF4, further promoting the expression of CHOP, thereby leading to neuronal injury and death [39, 40]. Previous studies have confirmed that ER stress plays an important role in neuronal injury and the pathogenesis of neurodegenerative diseases [15, 18, 19, 41]. In order to confirm whether ER stress-associated PERK pathway plays a role in stress-induced degeneration and death of glutamatergic neurons, we used a specific inhibitor Sal. Sal protects cells by selectively inhibiting the dephosphorylation of p-eIF2*α* in the PERK pathway [42, 43]. Our results showed that Sal could reduce the damage of glutamatergic neurons by downregulating the expression of ATF4 and CHOP, improve the glutamatergic neuronal function, and alleviate the psycho-behavioral disorders. Therefore, 20377722D the ER-PERK pathway is one of the crucial pathomechanisms for stress-induced glutamatergic neuronal death in the BLA nucleus of the amygdala.

## 5. Conclusion

Chronic stress can lead to the degeneration and death of glutamatergic neurons in the BLA nucleus, resulting in psychobehavioral disorders and ER stress. Moreover, the ER stress-associated PERK pathway may be one of the key mechanisms in stress-induced glutamatergic neuronal degeneration and death in the amygdala.

## Data Availability

The data used to support the findings of this study are included within the article.

## References

[1] Yaribeygi H., Panahi Y., Sahraei H., Johnston T. P., Sahebkar A. (2017). The impact of stress on body function: a review. *EXCLI Journal*.

[2] Kivimäki M., Steptoe A. (2018). Effects of stress on the development and progression of cardiovascular disease. *Nature Reviews Cardiology*.

[3] Ginty A. T., Kraynak T. E., Fisher J. P., Gianaros P. J. (2017). Cardiovascular and autonomic reactivity to psychological stress: neurophysiological substrates and links to cardiovascular disease. *Autonomic Neuroscience*.

[4] Peña-Bautista C., Casas-Fernández E., Vento M., Baquero M., Cháfer-Pericás C. (2020). Stress and neurodegeneration. *Clinica Chimica Acta*.

[5] Kruk J., Aboul-Enein B. H., Bernstein J., Gronostaj M. (2019). Psychological stress and cellular aging in cancer: a meta-analysis. *Oxidative medicine and cellular longevity*.

[6] Yang T. T., Simmons A. N., Matthews S. C. (2010). Adolescents with major depression demonstrate increased amygdala activation. *Journal of the American Academy of Child and Adolescent Psychiatry*.

[7] Ding Y., Dai J. (2019). Advance in stress for depressive disorder. *Advances in Experimental Medicine and Biology*.

[8] Shin L. M., Liberzon I. (2010). The neurocircuitry of fear stress and anxiety disorders. *Neuropsychopharmacology*.

[9] Sheline Y. I., Barch D. M., Donnelly J. M., Ollinger J. M., Snyder A. Z., Mintun M. A. (2001). Increased amygdala response to masked emotional faces in depressed subjects resolves with antidepressant treatment: an fMRI study. *Biological Psychiatry*.

[10] Sharp B. M. (2017). Basolateral amygdala and stress-induced hyperexcitability affect motivated behaviors and addiction citation. *Translational psychiatry*.

[11] Davis M., Whalen P. J. (2001). The amygdala: vigilance and emotion. *Molecular Psychiatry*.

[12] Pape H. C., Pare D. (2010). Plastic synaptic networks of the amygdale for the acquisition, expression and extinction of conditioned fear. *Physiological Reviews*.

[13] Prager E. M., Bergstrom H. C., Wynn G. H., Braga M. F. (2016). The basolateral amygdale gamma-aminobutyric acidergic system in health and disease. *Journal of Neuroscience Research*.

[14] Sah P., Faber E. S. L., Lopez de Armentia M., Power J. (2003). The amygdaloid complex: anatomy and physiology. *Physiological Reviews*.

[15] Wang S., Shi W., Zhang G. (2019). Endoplasmic reticulum stress-mediated basolateral amygdala GABAergic neuron injury is associated with stress-induced mental disorders in rats. *Frontiers in cellular neuroscience*.

[16] Lowery-Gionta E. G., Crowley N. A., Bukalo O., Silverstein S., Holmes A., Kash T. L. (2018). Chronic stress dysregulates amygdalar output to the prefrontal cortex. *Neuropharmacology*.

[17] Popoli M., Yan Z., BS M. E., Sanacora G. (2012). The stressed synapse: the impact of stress and glucocorticoids on glutamate transmission. *Nature Reviews Neuroscience*.

[18] Yi S. Y., Shi W., Wang H. (2017). Endoplasmic reticulum stress PERK-ATF4-CHOP pathway is associated with hypothalamic neuronal injury in different durations of stress in rats. *Frontiers in Neuroscience*.

[19] Yi S. Y., Chen K., Zhang L. (2019). Endoplasmic reticulum stress is involved in stress-induced hypothalamic neuronal injury in rats via the PERK-ATF4-CHOP and IRE1-ASK1-JNK pathways. *Frontiers in Cellular Neuroscience*.

[20] Scholl J. L., Afzal A., Fox L. C., Watt M. J., Forster G. L. (2019). Sex differences in anxiety-like behaviors in rats. *Physiology & Behavior*.

[21] Novaes L. S., dos Santos N. B., Batalhote R. F. P. (2017). Environmental enrichment protects against stress-induced anxiety: Role of glucocorticoid receptor, ERK, and CREB signaling in the basolateral amygdala. *Neuropharmacology*.

[22] de Oliveira Citó Mdo C., da Silva F. C., Silva M. I. (2012). Reversal of cocaine withdrawal-induced anxiety by ondansetron, buspirone and propranolol. *Behavioural Brain Research*.

[23] Paxinos G., Watson C. (2007). *The Rat Brain in Stereotaxic Coordinates*.

[24] Chen Y. P., Wang C., Xu J. P. (2019). Chronic unpredictable mild stress induced depression- like behaviours and glutamate-glutamine cycling dysfunctions in both blood and brain of mice. *Pharmaceutical Biology*.

[25] Hamzei Taj S., Kho W., Aswendt M. (2016). Dynamic modulation of microglia/macrophage polarization by miR-124 after focal cerebral ischemia. *Journal of Neuroimmune Pharmacology*.

[26] Larry C. S., Keri J. H. (2000). Fluoro-Jade B: a high affinity fluorescent marker for the localization of neuronal degeneration. *Brain Research*.

[27] Lucassen P. J., Pruessner J., Sousa N. (2014). Neuropathology of stress. *Acta Neuropathologica*.

[28] Hetzel A., Rosenkranz J. A. (2013). Distinct effects of repeated restraint stress on asolateral amygdala neuronal membrane properties in resilient adolescent and adult rats. *The International Journal of Neuropsychopharmacology*.

[29] Hertz L., Peng L., Dienel G. A. (2007). Energy metabolism in astrocytes: high rate of oxidative metabolism and spatiotemporal dependence on glycolysis/glycogenolysis. *Journal of Cerebral Blood Flow and Metabolism*.

[30] Choi H. B., Gordon G. R., Zhou N. (2012). Metabolic Communication between Astrocytes and Neurons via Bicarbonate- Responsive Soluble Adenylyl Cyclase. *Neuron*.

[31] Fu X., Wu H., Li J. (2017). Efficacy of drug interventions for chemotherapy-induced chronic peripheral neurotoxicity: a network meta-analysis. *Frontiers in neurology*.

[32] Cassar M., Issa A. R., Riemensperger T. (2015). A dopamine receptor contributes to paraquat-induced neurotoxicity in drosophila. *Human Molecular Genetics*.

[33] Ahmed-Leitao F., Spies G., van den Heuvel L., Seedat S. (2016). Hippocampal and amygdala volumes in adults with posttraumatic stress disorder secondary to childhood abuse or maltreatment: a systematic review. *Psychiatry Research: Neuroimaging*.

[34] Dong L., Wang S., Li Y. (2017). RU486 reverses emotional disorders by influencing astrocytes and endoplasmic reticulum stress in chronic restraint stress challenged rats. *Cellular Physiology and Biochemistry*.

[35] Vishnoi S., Raisuddin S., Parvez S. (2016). Glutamate excitotoxicity and oxidative stress in epilepsy: modulatory role of melatonin. *Journal of environmental pathology, toxicology and oncology*.

[36] Satoh E., Tada Y., Matsuhisa F. (2011). Chronic stress enhances calcium mobilization and glutamate exocytosis in cerebrocortical synaptosomes from mice. *Neurological Research*.

[37] Lu M., Lawrence D. A., Marsters S. (2014). Opposing unfolded-protein-response signals converge on death receptor 5 to control apoptosis. *Science*.

[38] Obakan-Yerlikaya P., Arisan E. D., Coker-Gurkan A. (2017). Calreticulin is a fine tuning molecule in epibrassinolide-induced apoptosis through activating endoplasmic reticulum stress in colon cancer cells. *Molecular Carcinogenesis*.

[39] Marciniak S. J., Yun C. Y., Oyadomari S. (2004). CHOP induces death by promoting protein synthesis and oxidation in the stressed endoplasmic reticulum. *Genes & Development*.

[40] Novoa I., Zeng H., Harding H. P., Ron D. (2001). Feedback inhibition of the unfolded protein response by GADD34-mediated dephosphorylation of eIF2alpha. *The Journal of Cell Biology*.

[41] Ho Y. S., Yang X., Lau J. C. (2012). Endoplasmic Reticulum Stress Induces Tau Pathology and Forms a Vicious Cycle: Implication in Alzheimer's Disease Pathogenesis. *Journal of Alzheimer’s disease*.

[42] Boyce M., Bryant K. F., Jousse C. (2005). A selective inhibitor of eIF2*α* dephosphorylation protects cells from ER stress. *Science*.

[43] Yuan Y. (2019). Alpha-lipoic acid protects against cadmium-induced neuronal injury by inhibiting the endoplasmic reticulum stress eIF2a-ATF4 pathway in rat cortical neurons in vitro and in vivo. *Toxicology*.

